# Distinct effects of progesterone and cholesterol on lipid membranes: insights from biophysical experiments and molecular dynamics simulations

**DOI:** 10.3389/fmolb.2025.1662811

**Published:** 2025-10-13

**Authors:** Anna Lągowska, Emilia Krok, Maria Domanska, Piotr Setny, Lukasz Piatkowski, Hanna Orlikowska-Rzeznik

**Affiliations:** ^1^ Institute of Physics, Faculty of Materials Engineering and Technical Physics, Poznan University of Technology, Poznan, Poland; ^2^ Biomolecular Modelling Group, Centre of New Technologies, University of Warsaw, Warsaw, Poland

**Keywords:** progesterone, sex hormones, non-genomic steroid signalling, lipid bilayer, model cell membrane, membrane heterogeneity, lipid dynamics, cholesterol

## Abstract

Steroid hormones, including progesterone, are known to exert genomic, non-genomic and non-specific effects. However, their influence on membrane biophysics remains unclear. In this study, we investigate the distinct membrane-modulating behaviour of progesterone compared to cholesterol, employing a multidisciplinary approach that combines fluorescence microscopy, steady-state spectroscopy, and atomistic molecular dynamics simulations. Our results demonstrate that, whereas cholesterol promotes lipid packing and stabilises phase-separated domains, progesterone disrupts phase separation, reduces line tension and increases lipid lateral diffusion, without significantly altering local membrane fluidity. Molecular simulations reveal that progesterone is more variably oriented and distributed within the bilayer than cholesterol. This results in membrane thinning and differential ordering of lipid tails. These structural effects may lead to increased membrane permeability and dynamic reorganization, which could facilitate rapid non-genomic signalling. Notably, the effects of progesterone are more pronounced in multicomponent, phase-separated membranes than in homogeneous lipid systems, suggesting context-specific roles. Our findings present progesterone as a dynamic modulator of membrane organisation, with implications for hormone signalling, drug delivery and therapeutic action in pharmacological settings.

## 1 Introduction

Steroid hormones play a key role in the development of cells and organs, the physiological changes experienced during adolescence, the control of reproduction, the maintenance of systemic homeostasis, and the protection and regulation of cognitive health (Cole et al., 2019; García-Gómez et al., 2020). They have also found pharmacological applications in the treatment of a number of autoimmune diseases, as anti-inflammatory drugs, and in contraception (Orme et al., 1983; Ericson-Neilsen and Kaye, 2014). Their classical mode of action involves diffusion across the cell membrane into the cytoplasm, where they bind to intracellular steroid receptors. This leads to changes in gene expression, known as the genomic pathway (Beato et al., 1995). This mechanism exerts physiological responses that typically occur with latency periods of hours or days.

However, it is also known that steroid hormones can elicit rapid cellular responses (seconds to minutes) that cannot be explained by the genomic pathway (Cato et al., 2002). This non-classical, non-genomic pathway has been partially explained on the basis of the existence of membrane-embedded steroid receptors that, when activated by the binding of steroid hormone, trigger signalling cascades in the cell (Schwartz et al., 2016). Importantly, it has been found that steroid binding sites of certain receptors may be located within their intramembranous region. This suggests that steroids must first integrate into the lipid membrane to access the receptor docking site (Akk et al., 2009).

Interestingly, there are indications that, in addition to interacting specifically with receptors, steroid hormones also elicit a non-specific cellular response, for example, by intercalating into the cell membrane and altering its biophysical properties, such as fluidity and microdomain organisation (Dindia et al., 2013). This may lead to the modification of signalling pathways.

Cholesterol, a structural steroid and biosynthetic precursor of steroid hormones, is recognised for its crucial role in shaping the structure, dynamics, and function of eukaryotic cell membranes (Czub and Baginski, 2006). Cholesterol is well known to order lipid chains, promote tight lipid packing, and stabilise liquid-ordered domains in membranes (Kurad et al., 2004; Hung et al., 2007; Fritzsching et al., 2013; Kotenkov et al., 2019). In contrast, the biophysical effects of other steroids, including progesterone, are less well understood. A number of studies addressed this issue, yet the conclusions presented are far from unified. The wide variety of progesterone concentrations used in the literature probably reflects both methodological considerations (e.g., the sensitivity of the chosen technique) and variations in the biological or model membrane context being studied. In our study, we selected concentrations that are directly comparable to those used in cholesterol-containing membranes (10–50 mol%), and that are relevant to potential local steroid enrichment in biological membranes.

For example, Korkmaz and Severcan (2005), reported that low concentrations of progesterone (3 and 6 mol%) decrease membrane order (increase fluidity) in saturated phospholipid dipalmitoylphosphatidylcholine (DPPC) bilayers, regardless of the phase state in which it was present, while having negligible effects at higher concentrations. Abboud et al. (2015) observed a continuous increase in membrane fluidity with increasing progesterone concentration for the same membrane composition. In contrast, Whiting et al. (2000) found that progesterone decreased the fluidity in membranes composed of egg yolk phosphatidylcholine and native biological membranes across a wide range of concentrations (9–30 mol%). Using only 0.2 mol% of progesterone, (Liang et al., 2001) reported no significant effect on the fluidity of the PC-containing membrane.

Taken together, these reports suggest that progesterone’s impact on membrane fluidity is highly context-dependent, varying with lipid composition, phase state, and steroid molar fraction, rather than being universal. This variability underscores the importance of conducting systematic, side-by-side studies of multiple membrane compositions under well-controlled experimental and computational conditions.

It should be noted that McDonnel et al. (2003) showed in living cell studies that incubation of epithelial ovarian cancer cells in the presence of 100 ng/mL of progesterone reduces the fluidity of their plasma membranes. This translates into the inhibition of carcinogenesis. Therefore, it has been hypothesised that progestins (exogenous progesterone) may contribute to the prevention and/or treatment of early-stage ovarian cancer.

The control of lateral lipid mobility within the membrane is one of the key factors required for the membrane repair process, as plasma membrane damage must be repaired quickly to avoid cell death (Horn and Jaiswal, 2019). Some reports suggest that reducing the lateral mobility of lipids can measurably contribute to the repair of damaged cells, such as the muscle fibres of people with Limb Girdle Muscular Dystrophy 2B (LGMD2B). The cells of these individuals naturally exhibit increased membrane fluidity, which has been linked to impaired membrane repair in this disease (Sreetama et al., 2018). Sreetama et al., for example, showed that the conventional glucocorticoid prednisolone, which is the mainstay treatment for inflammatory muscle diseases, accelerates the lipid membrane diffusion to an undesired extent, thereby impeding membrane repair even further. In contrast, vamorolone, which is structurally closely related to prednisolone, stabilised the membrane of diseased muscle cells by decreasing the lateral lipid mobility. This resulted in diffusion rates similar to those of healthy muscle cells, thus improving the repair of dystrophic myofibres in LGMD2B patients. Moreover, to establish if membrane lipid mobility is unambiguously associated with membrane repair, they conducted a simple test using methyl beta cyclodextrin (MβCD), a known membrane fluidising agent, and concluded that the injured myoblasts cells’ ability to repair themselves following laser injury was indeed compromised under this influence (Sreetama et al., 2018). On the other hand, Van Bömmel and co-workers (Van Bommel et al., 1987) studied the impact of various steroid hormones on the lateral diffusion of a fluorescent lipid probe (NBD-PC) within the plasma membrane of intact breast cancer cells using the fluorescence recovery after photobleaching (FRAP) technique. They found that a high dose (10^−7^–10^−5^ mol/L) of medroxyprogesterone acetate (MPA), a synthetic progestin caused a significant decrease in the lateral diffusion coefficient of NBD-PC. The other steroids tested were significantly less effective or had no effect. It was concluded that the direct interaction of MPA with membranes may play a role in the compound’s antitumour activity (Van Bommel et al., 1987).

As shown, steroids can have a bidirectional effect on lipid diffusion, either accelerating it or slowing it. Given the duality of these mechanisms, lipid diffusion likely plays a context-dependent role in membrane repair. It is not obvious whether accelerating or slowing down the diffusion is advantageous, and each situation must be considered individually. However, since the available studies have primarily been performed on native cellular membranes, where many confounding factors are at play, a deeper mechanistic understanding is still lacking. Controlled studies using simplified model membranes will be essential to disentangle the direct contributions of steroids to lipid mobility and repair.

(Horn and Jaiswal, 2019; Sreetama et al., 2018; Van Bommel et al., 1987) Clearly, changes in the physicochemical properties of membranes and their cellular effects may have implications for clinical practice and drug design. Importantly, the impact of steroids on biological membrane properties may depend on the lipid membrane composition and the percentage of a specific steroid within it.

Altogether, these observations highlight the need for comprehensive investigations that simultaneously monitor multiple biophysical parameters of model membranes under well-controlled conditions. Key unresolved questions remain: Does progesterone integrate into lipid bilayers, and if so, to what extent and depth? How does its incorporation influence membrane properties such as fluidity, thickness, phase behaviour, and lateral organization? Addressing these questions is critical for understanding the broader physiological roles of progesterone and its membrane-mediated mechanisms of action.

Understanding how steroids interact with and modify membrane structure is important not only for elucidating their established physiological and pharmacological roles, but also for exploring rapid, non-genomic signalling pathways and therapeutic strategies where membrane modulation could influence receptor activation, membrane repair or domain organization (Dindia et al., 2013; Sreetama et al., 2018; Larder et al., 2025).

In this work, we aim to resolve these uncertainties through a multifaceted approach that combines fluorescence microscopy, steady-state spectroscopy, and atomistic molecular dynamics (MD) simulations. Our results show that, in contrast to cholesterol, progesterone increases lipid mobility, modestly redistributes bilayer order, and impairs phase separation in multicomponent membranes. These biophysical effects imply that progesterone could function as a dynamic regulator of membrane organization, potentially enabling rapid, non-genomic signalling, particularly in pharmacological scenarios involving elevated local hormone concentrations.

## 2 Materials and methods

### 2.1 Materials

1,2-dimyristoleoyl-sn-glycero-3-phosphocholine (DmOPC), egg yolk sphingomyelin (SM), 1,2-dioleoyl-sn-glycero-3-phosphocholine (DOPC) and cholesterol (Chol) were obtained from Avanti Polar Lipids (Alabaster, AL, USA). 6-dodecanoyl-2-dimethylaminonaphthalene (Laurdan), 1,2-dioleoyl-sn-glycero-3-phosphoethanolamine labelled with Atto 633 (Atto 633-DOPE), progesterone (Prog), calcium chloride (CaCl_2_), sodium hydroxide (NaOH), sodium chloride (NaCl) and chloroform (HPLC grade) were purchased from Merck KGaA (Darmstadt, Germany). The buffer reagent 4-(2-hydroxyethyl)piperazine-1-ethanesulfonic acid (HEPES PUFFERAN) was obtained from Carl Roth GmbH + Co., KG (Karlsruhe, Germany). All lipids and reagents were used as received, without additional purification. Ultrapure water used in the experiments was produced using a Labopol-Polwater water purification system (Kraków, Poland). Glass coverslips No. 0 were acquired from Paul Marienfeld GmbH & Co., KG (Lauda-Königshofen, Germany). Mica sheets for the fabrication of solid-supported lipid membranes were supplied by Shree GR Exports Private Limited (Kolkata, India). The optical adhesive Norland 68, activated by ultraviolet light, was purchased from Thorlabs Sweden AB (Mölndal, Sweden). Two-component silicone Elite Double 22 Fast was obtained from Zhermack (Badia Polesine, Italy).

### 2.2 Preparation of small unilamellar vesicles

**FIGURE 1:**
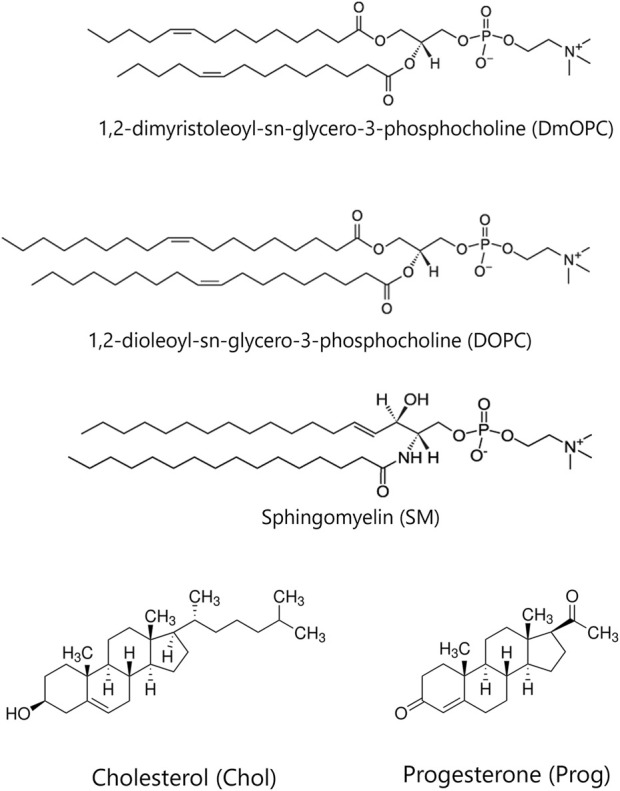
The chemical structures of the lipids and steroids used in the study.

Chloroform or chloroform:ethanol (in case of progesterone) stock solutions of: (i) DOPC, (ii) binary mixture of DmOPC with 30 mol% Prog, (iii) binary mixture of SM with 30 mol% Prog, (iv) binary mixtures of DOPC:Prog with Prog range 10–50 mol%, (v) binary mixtures DOPC:Chol with Chol range 10–50 mol%, (vi) equimolar binary mixture DmOPC:SM, (vii) equimolar ternary mixture DmOPC:SM:Prog and (viii) equimolar ternary mixture DmOPC:SM:Chol were prepared by mixing the desired ratio of lipids, maintaining 10 mM lipid concentration in each mixture. The molecular structures of the studied lipids and steroids are depicted in Figure 1. For imaging purposes Atto 633-DOPE and Laurdan were added, keeping the 1:1000 probe to lipid ratio. Lipid solutions mixed with fluorescent probes were evaporated under a gentle stream of nitrogen gas for 20 min, followed by further drying in the vacuum chamber for a minimum of 2 h to remove any traces of residual organic solvent. The dried lipid film was then rehydrated with buffer solution (10 mM HEPES, 150 mM NaCl, pH 7.4 adjusted with NaOH) to achieve a lipid concentration of 10 mM. To promote uniform dispersion, the suspension underwent four cycles of heating to 60 °C and vortexing, each step lasting 1 min, leading to the formation of multilamellar vesicles (MLVs). The lipid suspension was distributed into sterile glass vials (10 µL per vial) and stored at −20 °C until further use. The resulting MLVs suspension of the desired lipid composition was further diluted tenfold by adding 90 µL of HEPES buffer, resulting in a final lipid concentration of 1 mM. This mixture was then subjected to bath sonication (ultrasonic cleaning bath Bransonic^®^, CPXH1800-E, Connecticut, United States) for 10 min, until the solution became transparent, indicating the successful formation of small unilamellar vesicles (SUVs).

### 2.3 Preparation of solid-supported lipid bilayers

To prepare the solid support, a small droplet of immersion oil was applied to a No. 0 glass coverslip. A thin, freshly cleaved mica sheet, pre-cut into circular pieces with a diameter of 9.53 mm (3/8 inch),was then carefully placed onto the oil and secured by applying UV-cured adhesive around its edges. A half-cute Eppendorf tube, was positioned over the mica-covered coverslip and sealed with silicone, creating a water reservoir. 100 μL of the SUVs suspension was gently applied onto the mica, immediately followed by the addition of 2 µL of a 0.1 M CaCl_2_ solution. After approximately 30 s, 600 µL of the buffer was added to prevent drying of the bilayer. The sample was incubated for 30 min at room temperature (21 °C) to facilitate the rupture and spreading of vesicles over the mica surface. Subsequently, the supported lipid bilayer (SLB) was carefully rinsed 20 times with 1 mL of buffer (total 20 mL of buffer) to remove any unburst vesicles. The reservoir was then filled with buffer solution, covered with a glass coverslip and sealed with silicone. Some of the samples referred to as “heated” in the manuscript were prepared under elevated temperature conditions. In this case, SLBs preparation was performed on a hot plate set to 60 °C. Washing was carried out using pre-heated to 60 °C buffer to prevent a rapid drop in the sample’s temperature. The samples were then gradually cooled on the turned-off hot plate until reaching room temperature equilibrium.

### 2.4 Imaging systems

#### 2.4.1 Steady-state emission spectra acquisition

Experiments were carried out on a manually operated inverted microscope (Carl Zeiss, Axiovert 200). The excitation beam at 370 nm was delivered by a pulsed supercontinuum laser (NKT Photonics, SuperK FIANIUM FIU-15) equipped with a UV extension module (NKT Photonics, SuperK EXTEND-UV). All measurements were performed using nonpolarised excitation light. A 387 nm long-pass dichroic mirror (Chroma, T387LP) directed the excitation beam into an oil immersion objective (Carl Zeiss, EC Plan-Neofluar ×100/1.30), focusing it onto a diffraction-limited spot at the sample plane and allowing the emitted fluorescence to pass through to the detection system. The resulting epifluorescence signal was filtered using a 380 nm long-pass filter (Semrock, FF01-380/LP-25). For imaging, the fluorescence was directed to a single-photon counting module (Hamamatsu Photonics, C11202-100), while for spectral acquisition, it was sent to a spectrograph (Andor, Kymera 328I-C) fitted with a 150 lines/mm diffraction grating and detected by an electron-multiplying CCD camera (Andor, iXon 888 UCS-BB) cooled to −70 °C. A remotely operated mirror allowed switching between the two detection paths. Photon counts from the single-photon module were digitised using a data acquisition card (National Instruments, NI USB-6363). To acquire images, the sample was scanned across the stationary laser focus in the x–y plane using a piezoelectric nanopositioning stage (Mad City Labs, Nano-LPS200), controlled by a Nano-Drive 3 controller (Mad City Labs). Sample positioning and image reconstruction were managed through custom software written in LabVIEW. To minimise sample photobleaching, its illumination was synchronised with data acquisition via an optical beam shutter (Thorlabs, SHB1T).

#### 2.4.2 Fluorescence imaging

Confocal fluorescence imaging and fluorescence recovery after photobleaching (FRAP) experiments were performed using laser-scanning confocal microscope (Carl Zeiss, LSM 710) equipped with an oil immersion objective EC Plan-Neofluar ×40/1.30 NA. Excitation of Atto 633-DOPE was performed using a HeNe laser at a wavelength of 633 nm, and its emission was recorded in the range of 645–797 nm. Intensity was carefully adjusted during imaging to minimise photobleaching of the fluorescent dye.

For FRAP measurements, a circular region with a diameter of 10 µm was photobleached, and the subsequent fluorescence recovery was recorded over time. Lipid diffusion coefficients (D) were extracted by fitting the recovery kinetics using a modified Soumpasis model described by Equations 1, 2 (Soumpasis, 1983).

$$f\left(t\right)=a\cdot e^{\frac{2\tau_{D}}{t}}\left(I_{0}\left(\frac{2\tau_{D}}{t}\right)+I_{1}\left(\frac{2\tau_{D}}{t}\right)\right)+b$$ (1)

where

$$\tau_{D}=\frac{w^{2}}{4D}$$ (2)

where *a* represents the amplitude of the fitted recovery curve, *b* corresponds to the residual fluorescence after photobleaching, and *w* denotes the radius of the bleached region. *I*_*0*_ *(t)* and *I*_*1*_ *(t)* are the modified Bessel functions. The fitting procedure was applied to data normalised relative to the reference fluorescence intensity of the entire image, excluding the bleached region. The mobile fraction characteristic of the liquid-disordered phase was calculated according to Equation 3.

$$R_{mobile}=\frac{a}{1-b}$$ (3)

The parameters *a* and *b* were determined from the fitting procedure. Diffusion coefficients (*D*) were calculated for each FRAP trace and subsequently averaged over measurements obtained from at least 10 distinct regions within samples of a given steroid concentration.

To estimate the average size of lipid domains, original confocal images were processed by converting them into black-and-white binary images through contrast threshold adjustment in Fiji/ImageJ software. Images were smoothed, by replacing each pixel with the average value of its 3 × 3 neighbouring pixels (Schneider et al., 2012). A minimum of four different regions were selected from two independent samples of identical composition, resulting in a total of 16 images, 50 × 50 µm each.

To provide a quantitative measure of phase separation and the lateral organisation of lipid membranes, we calculated the Shannon entropy $S_{H}$ defined as depicted in Equation 4 (Shannon et al., 1949):

$$S_{H}=-\sum P_{x}\;\log\;P_{x}$$ (4)

where $P_{x}$ is the probability of finding domains belonging to size bin *x*.

Circularity was calculated for the phase-separated bilayers: DmOPC:SM, DmOPC:SM:Prog, and DmOPC:SM:Chol using Fiji/ImageJ software package, according to Equation 5 (Schindelin et al., 2012).

$$circularity=4\pi \frac{area}{{perimeter}^{2}}$$ (5)

### 2.5 Molecular dynamics simulations

We considered three kinds of membrane systems: (a) a one component bilayer, composed of DOPC molecules (120 lipids per monolayer), (b) a two component bilayer composed of DOPC and Chol molecules (90:30 lipids per monolayer), and (c) a two component bilayer composed of DOPC and Prog molecules (90:30 lipids per monolayer). The systems were assembled using CHARMM-GUI server (Jo et al., 2008) and parameterised using Amber Lipid21 (Dickson et al., 2022) for DOPC and Chol, Amber GAFF (Atkovska et al., 2018) for Prog, and TIP3P model (Jorgensen et al., 1983) was used for water. The systems were simulated at temperature 298 K maintained with Nosé–Hoover thermostat, and pressure 1 bar maintained with Parrinello-Rahman barostat, under periodic boundary conditions with default equilibration protocol provided by CHARMM-GUI followed by production runs of 0.5 μs. Simulations represented symmetric bilayers of (a) (8.9 nm × 8.9 nm), (b) (8.1 nm × 8.1 nm), and (c) (8.6 nm × 8.6 nm) in the XY plane, composed of lipid types used in the experiments, embedded in aqueous environment, each with 2.25 nm margin of water on both membrane sides. All simulations were carried out with Gromacs 2021 (Abraham et al., 2015; Lindahl, 2021) software. Lipid density profiles across the membrane and theta angles distribution were calculated using MDAnalysis python package (Michaud-Agrawal et al., 2011). The analysis was based on the last 100 ns of each molecular dynamics simulation.

To compute the theta (Θ) angle between the Z-axis and the vector defined by two atoms located at the ends of steroid rings, we used the C3 and C16 atoms from Chol and Prog, according to the IUPAC numbering. To determine the partial densities of the centre of the steroid rings along the Z-axis, we chose the centre of mass (COM) of the following atoms: C3, C8, C10, C13 and C16 for Chol and Prog, according to the IUPAC numbering. These atoms were selected as they span the rigid scaffold, which is common to both cholesterol and progesterone.

Diffusion coefficient for lateral lipid diffusion of DOPC was obtained from the mean square displacement (MSD) of the phosphate atoms, gathered over 100 ns and performing a linear fitting of the MSD data on the time interval between 30 ns and 70 ns. Mean square of the translation distance of the particle is proportional to 4 × D × t. Both MSD and D were calculated by the program gmx msd (Lindahl, 2021).

### 2.6 Lipid order parameter determination

The ordering of the lipid acyl chains was determined by calculation of the carbon-hydrogen bond order parameter (S_CH_). S_CH_ is a measure of the average relative orientation of the carbon-hydrogen bonds with respect to the bilayer normal. It was calculated according to the formula described in Equation 6.

$$S_{CH}=\frac{1}{2}\langle 3\cos^{2}\theta -1\rangle$$ (6)

In which ϕ is the angle between the bilayer normal and the vector joining carbon atom to its covalently bonded hydrogen atom, and   represents an ensemble average. This definition is directly analogous to the deuterium order parameter (S_CD_) determined in NMR spectroscopy (Seelig, 1977). Calculation of the order parameter of lipid acyl chains was carried out with g_lomepro software (Gapsys et al., 2013). The Error bars correspond to the standard error of the mean estimated based on the block averaging of 5 consecutive trajectory segments from the final 100 ns of the simulation.

### 2.7 Area per lipid determination

In a lipid membrane, lipids occupy space on the 2D plane of the bilayer. Voronoi tessellation partitions the plane into non-overlapping polygons, each surrounding one lipid such that any point inside a given cell is closer to that lipid’s reference point than to any other lipid’s reference point (Lukat et al., 2013). To this end, we represented each steroid by the oxygen atom bound to the C3 carbon atom (which is structurally equivalent in cholesterol and progesterone), and projected its xy position onto the membrane plane for Voronoi tessellation. This approach was adopted to minimise systematic differences between the two steroids and to position the representative point close to the lipid–water interface (similar to the phosphorus reference point used for phospholipids). The area of each Voronoi cell was taken as the area “belonging” to that lipid. To get the average area per lipid (APL) class, one has to average over all APL values of lipids belonging to one class (e.g., DOPC or Chol/Prog), over all simulation frames. To obtain the average APL for the whole system, the average over all APL values of all lipids, over all simulation frames has to be calculated. Standard deviation values were calculated for all APL values of a given class. The calculations were carried out using LiPyPhilic package (Smith and Lorenz, 2021).

## 3 Results

### 3.1 Membrane lateral structure

**FIGURE 2:**
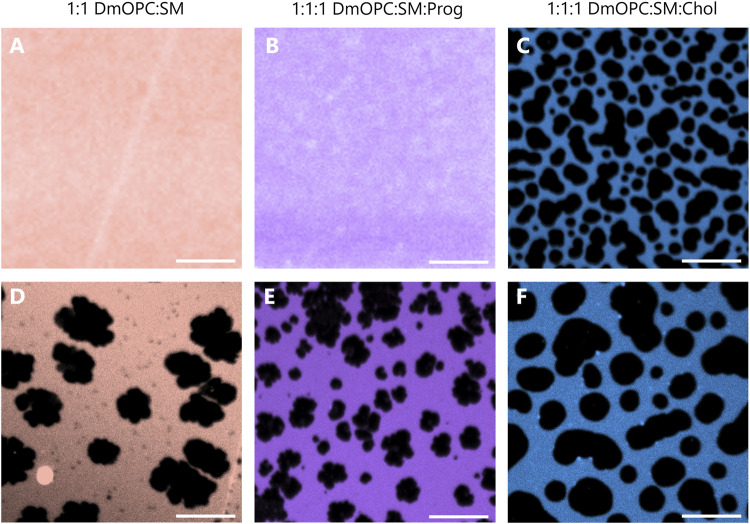
Representative confocal fluorescence microscopy images of mica-supported lipid bilayers composed of an equimolar mixture of DmOPC:SM **(A,D)**, DmOPC:SM:Prog **(B,E)**, and DmOPC:SM:Chol **(C,F)** prepared under two different temperature conditions. The top row shows samples prepared at ambient temperature (21 °C), while the bottom row shows samples prepared with the modifications as described in the *Materials and methods section*. Fluorescence imaging was performed at an ambient temperature (21 °C). The L_D_ phase is labeled with Atto 633-DOPE. The scale bar corresponds to 10 μm.

Representative fluorescence images of the samples prepared under ambient conditions are shown in Figures 2A–C, corresponding to equimolar mixtures of DmOPC:SM, DmOPC:SM:Prog, and DmOPC:SM:Chol, respectively. Among these, microscopic phase separation is observed only in the cholesterol-containing sample (Figure 2C), where distinct liquid-ordered (L_O_) phase domains are visible, surrounded by a continuous liquid-disordered (L_D_) phase region.

Despite the structural similarity between cholesterol and progesterone–both molecules containing a steroid core–the absence of microscopic phase separation in the progesterone-containing membrane (Figure 2B) suggests a distinct mode of interaction between these steroids and phospholipids.

To promote potential phase separation in the cholesterol-free systems, we applied heat at different stages of the sample preparation protocol (see the Materials and methods section for details). Following this treatment, temperature-induced phase separation was observed in both the DmOPC:SM and DmOPC:SM:Prog samples (Figures 2D,E), with domain structures forming at a micrometre scale. This behaviour is attributed to the selective phase transition of SM upon cooling: as SM is a higher-melting lipid, it diffuses and incorporates into growing domains through mechanisms governed by molecular mobility and energetic favourability (Szmodis et al., 2010).

In the DmOPC:SM:Chol sample, heating also affected membrane morphology. After thermal treatment (Figure 2F), L_O_ domains appeared larger and more widely spaced, separated by extended L_D_ regions compared to the unheated condition (Figure 2C). Interestingly, the lateral organisation of the membranes varies significantly among the three compositions. The DmOPC:SM membrane exhibits lateral segregation into SM-rich gel phase (S_O_) domains surrounded by a fluid L_D_ phase, which is formed predominantly by unsaturated DmOPC lipids. Most of the sphingomyelin molecules are concentrated in large, patchy areas, referred to as domains (Figure 2D). Notably, numerous small dark spots are visible near the S_O_ domains, which may represent nascent domains or residual SM fractions remaining within the L_D_ phase. In the DmOPC:SM:Prog sample (Figure 2E), there is greater heterogeneity in domain shape and size. Overall, the domains are smaller and less jagged than those observed in the DmOPC:SM membrane. However, based on morphology alone, the phase state of these domains cannot be conclusively determined.

To quantify differences in membrane organisation, we calculated the Shannon entropy, a metric previously applied to quantify lipid mixing and phase separation effects in lipid membranes (Brandani et al., 2013; Liu et al., 2021; Saar et al., 2021). Shannon entropy was calculated based on the distributions of domain sizes in phase-separated membranes (see Supplementary Figure S1; Supplementary Table S1). The entropy was about 30% higher for the progesterone-containing membrane compared to the cholesterol-containing membrane ( $S_{H\_Prog}$ = 8.52 vs. $S_{H\_Chol}$ = 6.56), reflecting greater heterogeneity in domain size for the former membrane.

**FIGURE 3:**
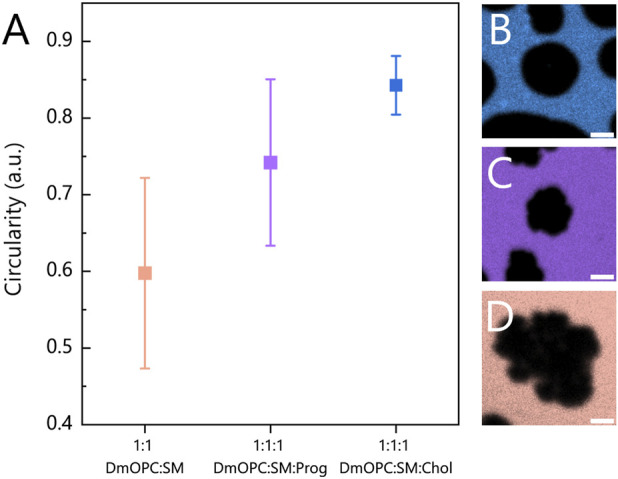
**(A)** Circularity analysis of domains (S_O_ or L_O_ phases) in heated mica-supported lipid bilayers composed of an equimolar mixture of: **(B)** DmOPC:SM:Chol, **(C)** DmOPC:SM:Prog and **(D)** DmOPC:SM. Each data point represents the circularity coefficient averaged over individual domains and calculated using the equation described in the Materials and methods section and averaged over at least two distinct samples per composition. The number of analysed domains per condition ranges from 116 to 353. Only domains clearly above the diffraction limit (i.e., with a diameter of over 3 μm) were included in the analysis. The error bars indicate the standard deviation of the domain circularity measurements. The scale bar corresponds to 2 μm.

Furthermore, to quantify the differences in domain morphology across the three compositions, we determined the circularity of micrometre-sized domains, as illustrated in Figure 3A.

In the DmOPC:SM sample, the domains that are characteristic of the S_O_ phase, composed predominantly of saturated SM, exhibit the lowest circularity values and a broad distribution (Figure 3D). These irregular, jagged domain morphologies are consistent with previous reports (Leonenko et al., 2004; Nasrallah et al., 2019; Garvey et al., 2023). In the presence of progesterone (Figure 3C), the domains appear smaller and more circular. However, the distribution of the circularity parameter remains broad, similar to that observed in the DmOPC:SM membrane.

The circularity of the analysed domains indicates that progesterone undoubtedly influences the phase separation process, but in a manner that is distinct from that of cholesterol. Progesterone appears to significantly reduce the line tension at the phase boundary compared to cholesterol-containing membranes. When line tension is high, domains tend to adopt more circular shapes, as observed in the DmOPC:SM:Chol sample (Figure 3B). These observations support two key conclusions: first, that progesterone partitions into the membrane, and second, that it affects the phase separation behaviour of lipid mixtures.

### 3.2 Membrane fluidity

Given the clear impact of progesterone on the lateral structure of membranes, it was essential to determine whether it also alters their physicochemical properties, such as fluidity. To address this, we employed the solvatochromic probe Laurdan to assess fluidity across different membrane environments in a spatially resolved manner. Laurdan fluorescence is highly sensitive to local polarity, i.e., hydration level (Salvolini et al., 2013), and dipolar relaxation dynamics near its fluorophore site, which is located just beneath the glycerol backbone of phospholipids (Jurkiewicz et al., 2012). As such, shifts in Laurdan’s emission spectrum provide insights into membrane fluidity: a red shift indicates increased fluidity, whereas a blue shift corresponds to a more ordered/rigid environment (Orlikowska-Rzeznik et al., 2023).

**FIGURE 4:**
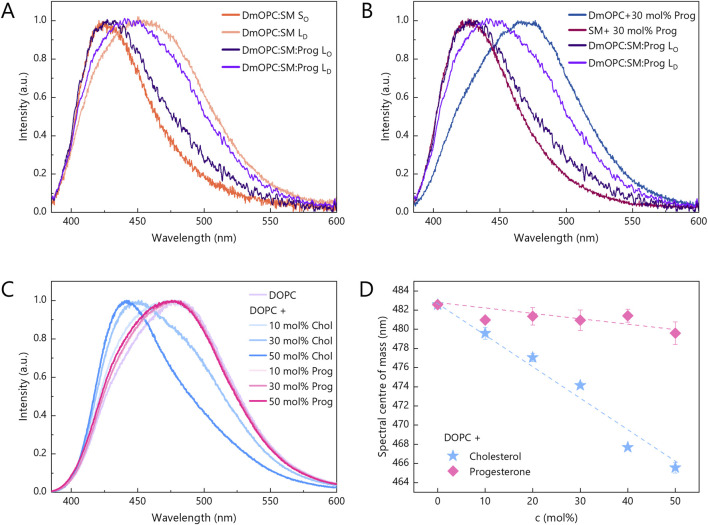
Changes in the fluorescence spectrum of Laurdan embedded in lipid bilayers in response to: **(A,B)** different membrane compositions and **(C)** an increasing amount of progesterone and cholesterol, ranging from 0 mol% to 50 mol%. Intermediate amounts include 10 mol% and 30 mol%. Laurdan spectra for progesterone and cholesterol contents of 20 mol% and 40 mol% are shown in Supplementary Figure S2. The spectra for each composition and steroid content were averaged from two independent samples and normalised. At least 27 and 12 spectra from distinct areas were analysed for single phase and phase-separated samples, respectively. All spectra were acquired at ambient temperature (21 °C). **(D)** Centre of mass of Laurdan spectra as a function of progesterone (pink diamonds) and cholesterol (blue stars) mole percentage. Error bars indicate standard deviations.

Figure 4A shows the Laurdan fluorescence spectra acquired from both the SM-rich domains and the surrounding L_D_ phase in the DmOPC:SM (orange shades) and DmOPC:SM:Prog (violet shades) samples. In the DmOPC:SM membrane, the Laurdan spectra reveal a significant phase contrast: the SM-rich domains exhibit a peak near 425 nm, which is characteristic of a rigid gel phase. In contrast, the L_D_ phase shows a red-shifted spectrum, which is indicative of a more fluid environment. Upon the addition of progesterone, this spectral contrast diminishes. The SM-rich domains exhibit a slight red shift (indicating increased fluidity), whereas the L_D_ phase shows a small blue shift (suggesting a subtle decrease in fluidity). This bilateral shift resembles the effect observed for cholesterol (Supplementary Figure S2A). The spectral centres of mass for all spectra shown in the main text and Supplementary Material are summarised in Supplementary Tables S1–S3.

These results clearly demonstrate that progesterone modulates the fluidity of phase-separated membranes. However, this effect could be due to one of two distinct mechanisms: (A) progesterone alters membrane fluidity directly through intermolecular interactions with phospholipids, or (B) progesterone impedes lipid phase separation, thereby altering the global fluidity distribution.

To distinguish between these mechanisms, we next examined membranes composed of a single phospholipid species, lacking phase separation, focusing on assessing the direct effect of progesterone. In unsaturated DmOPC membranes containing 30 mol% progesterone, the Laurdan spectrum was markedly red-shifted compared to the L_D_ phase in the DmOPC:SM:Prog sample (Figure 4B), indicating enhanced fluidity. Conversely, the Laurdan spectrum in SM membranes containing 30 mol% of progesterone was blue-shifted relative to the SM-rich domains in the DmOPC:SM:Prog sample (Figure 4B), indicating increased rigidity. These observations demonstrate significant spectral differences between the two monophasic systems, suggesting that progesterone’s direct impact on membrane fluidity is rather negligible.

To further investigate this, we examined the effect of varying progesterone concentrations on Laurdan emission in DOPC membranes, which are particularly susceptible to steroid-induced changes due to their inherent disorder. For comparison, we also assessed the effect of cholesterol (Figure 4C). Cholesterol caused a progressive and substantial blue shift of the Laurdan spectra (Supplementary Figure S2B), which is indicative of strong membrane stiffening. In contrast, progesterone had only a subtle impact, even at concentrations of up to 50 mol% (Supplementary Figure S2C). Figure 4D illustrates this contrast, showing that the spectral centre of mass shifts by ∼16 nm for 50 mol% cholesterol, but by only ∼2 nm for the same concentration of progesterone.

Together, these results confirm that progesterone has a negligible direct impact on the fluidity of homogenous membranes, whether composed of either saturated or unsaturated lipids. Its effect is only pronounced in multicomponent systems exhibiting phase separation. This strongly suggests that the changes observed in DmOPC:SM:Prog membranes are not due to specific lipid-progesterone interactions, but rather to progesterone’s ability to disrupt phase separation. This leads to a reduced contrast in fluidity between coexisting membrane regions. This mechanism differs from that of cholesterol, which promotes domain formation and enhances membrane heterogeneity.

### 3.3 Lipid lateral diffusion

Although Laurdan spectroscopy reveals how progesterone influences local lipid hydration and/or dipolar relaxation dynamics, it does not capture the broader, collective behaviour of lipids within the membrane. To assess whether progesterone affects long-range lipid mobility—and how this compares to the established effect of cholesterol—we performed fluorescence recovery after photobleaching (FRAP) measurements on pure DOPC membranes and binary mixtures containing either progesterone or cholesterol.

**FIGURE 5:**
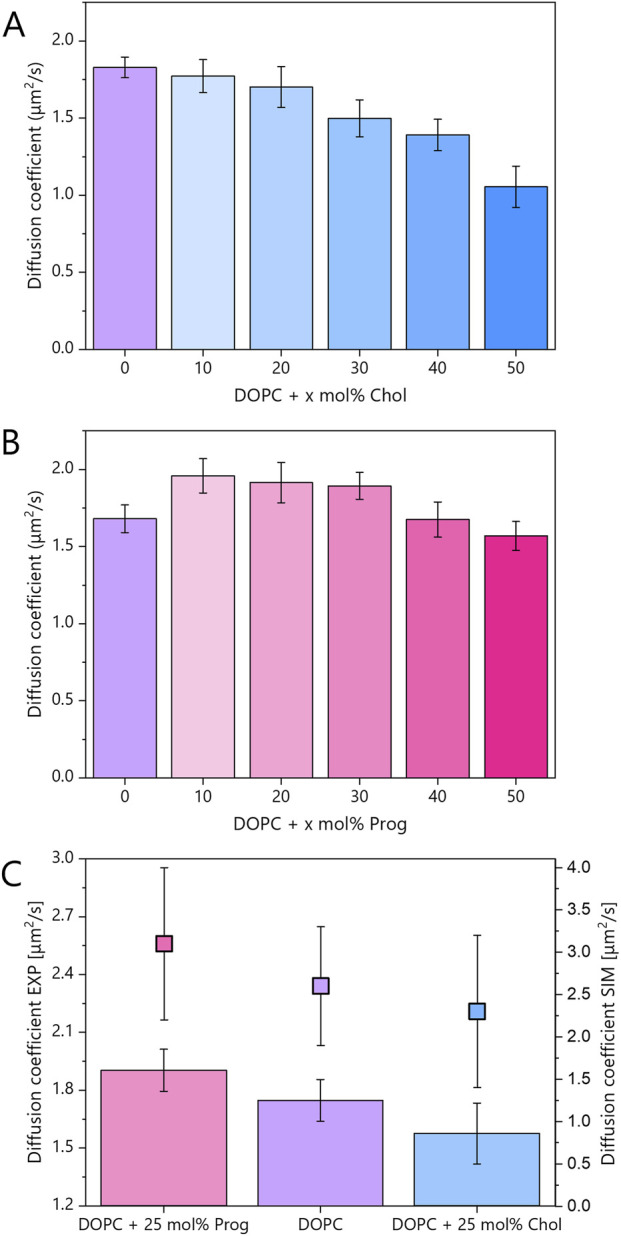
**(A,B)** Diffusion coefficients for mica-supported lipid bilayers composed of pure DOPC and its binary mixtures with the steroid molecules: cholesterol and progesterone, at varying molar concentrations. Error bars indicate standard deviations. FRAP traces are shown in Supplementary Figure S3 and mobile fraction in Supplementary Figure S4. **(C)** Diffusion coefficients for mica-supported lipid bilayers composed of pure DOPC and binary mixtures containing 25 mol% progesterone or cholesterol. The experimental data (bars, left axis) represent the average of at least 20 FRAP traces per composition, collected from two independent samples. These data were averaged from samples containing 20 and 30 mol% of steroids, within the L_D_ phase, which was labelled with Atto 633-DOPE. The data points (filled squares, right axis) correspond to the diffusion coefficients obtained from MD simulations (see Supplementary Figure S5). The error bars indicate the standard deviations in the case of the experimental data and the fitting error of the slope of the linear function in the case of the MD simulations.

FRAP recovery curves for varying steroid concentrations are presented in Supplementary Figure S3, while corresponding diffusion coefficients are summarised in Figure 5.

The average experimental diffusion coefficient for pure DOPC was 1.74 μm^2^/s. As expected, cholesterol reduced lipid mobility in a monotonic fashion. In contrast, and somewhat unexpectedly, progesterone displayed a biphasic behaviour (see Figure 5B). At intermediate concentrations (10–30 mol%), in DOPC:Prog samples, lipid diffusion increased relative to pure DOPC, but at higher concentrations (40–50 mol%), the diffusion coefficient returned to values similar to, or slightly lower than, those of pure DOPC.

We also evaluated the proportion of fluorescent lipids that can freely diffuse within the membrane, called mobile fraction, and found no significant changes across conditions (Supplementary Figure S4). This suggests that the observed effects reflect changes in diffusion dynamics rather than lipid immobility. In other words, the formation of immobile lipid accumulations is highly improbable. Consequently, there are no obstacles to the diffusion of free lipids.

In Figure 5C, experimental results from 20 to 30 mol% samples were averaged to align with MD simulations performed at 25 mol%. The diffusion coefficients from MD simulations were obtained by fitting the mean square displacement (MSD) of phosphorus atoms (see Supplementary Figure S5). The average diffusion coefficient derived from theoretical studies for pure DOPC was 2.6 μm^2^/s, higher than the experimental value due to the absence of substrate effects. Nevertheless, the relative trends were consistent: cholesterol slowed down lipid diffusion by ∼10% in experiment (1.58 μm^2^/s) and ∼12% in simulation (2.3 μm^2^/s), while progesterone enhanced diffusion by ∼9% in experiment (1.9 μm^2^/s) and ∼19% in simulation (3.1 μm^2^/s). The larger effect predicted in simulations likely reflects the absence of substrate interactions, which are known to restrict lipid motion in supported bilayers. Indeed, diffusion rates in free-standing bilayers are significantly higher than in supported systems (Przybylo et al., 2006). However, this effect is uniform across both leaflets (Zhang and Granick, 2005), and the key point is that relative trends, rather than absolute values, remain valid across systems and techniques.

The observed decrease in cholesterol’s fluidity (via Laurdan) and diffusivity (via FRAP) aligns with its ability to order and densify membranes (Warschawski and Devaux, 2005; Kučerka et al., 2008; Alwarawrah et al., 2010; Antila et al., 2022). In contrast, progesterone produced a modest reduction in the fluidity of the L_D_ phase but a measurable increase in overall diffusion. This combination suggests a fundamentally different mode of interaction with lipid bilayers.

### 3.4 Steroid positioning

**FIGURE 6:**
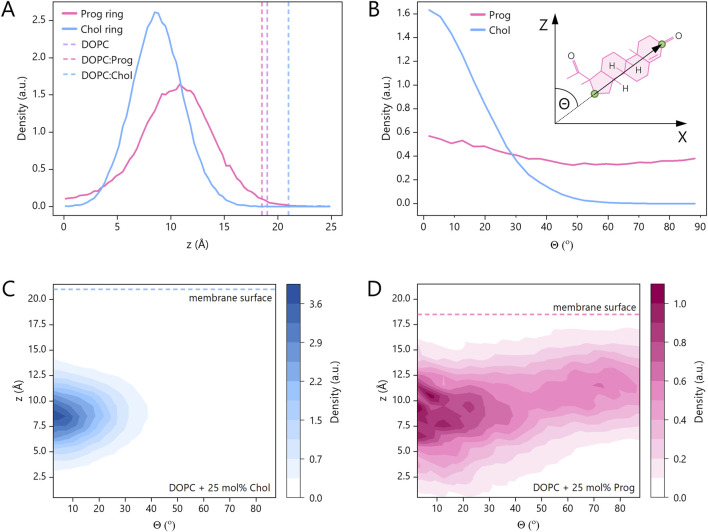
Steroid ring positions and orientations in systems containing DOPC and 25 mol% progesterone (pink) or 25 mol% cholesterol (blue) based on MD simulations. **(A)** Distribution of steroid ring centres along the membrane normal (Z-axis) relative to the bilayer midplane (Z = 0). The vertical dashed lines denote the membrane surface, which is defined as the point of maximum phosphorus atom density along the Z-axis. The membrane thickness, including both leaflets, is as follows: DOPC:Chol = (41.6 ± 0.2) Å, DOPC = (38.3 ± 0.1) Å and DOPC:Prog = (36.5 ± 0.3) Å. **(B)** Distribution of tilt angles (θ) between the long axis of the steroid core and the Z-axis for progesterone and cholesterol. **(C,D)** Joint probability distributions for tilt angles and ring positions with respect to the Z-axis.

To better understand the complex effects of progesterone observed in experiments, and building on the initial insights from MD simulations related to diffusion, we analysed its spatial distribution and orientation further. First, we analysed progesterone and cholesterol mass density profiles relative to the membrane normal (Figures 6A,C,D). Cholesterol exhibited a relatively narrow distribution across the membrane depth, with its centre of mass positioned at approximately 8 Å from the bilayer midplane, demonstrating no apparent tendency to reach either the membrane surface or its centre. In contrast, progesterone displayed a broader distribution, with a maximum shifted towards the membrane surface by ∼3 Å compared to cholesterol, as well as non-zero density in the membrane centre. As previously stated, the addition of cholesterol thickens the DOPC bilayer (Alwarawrah et al., 2010), which is consistent with our simulations. However, we observed the opposite effect with the progesterone molecule. As Larder et al. recently reported (Larder et al., 2025), progesterone thins the membrane, however, previous studies analysed the POPC molecule. Our data clearly sheds a light on the influence of progesterone on unsaturated lipids.

**FIGURE 7:**
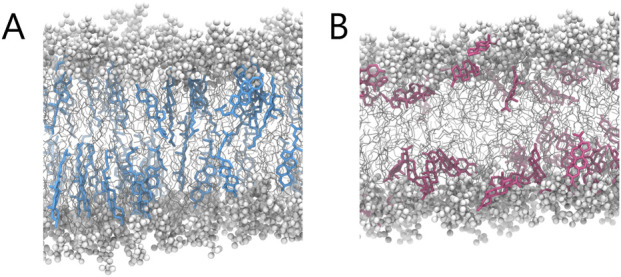
Representative MD snapshots illustrating the qualitative differences in molecular organisation between **(A)** cholesterol- and **(B)** progesterone-containing membrane.

Further differences were revealed by orientation analysis (see Figures 6B–D). Cholesterol predominantly adopted an upright position, with the plane of its rigid steroid core aligned perpendicular to the membrane surface and the hydroxyl group facing the aqueous environment. Progesterone, on the other hand, adopted a broader range of orientations, which also included a considerable fraction of membrane-parallel alignments. Interestingly, the latter were slightly more prevalent among molecules that were closer to the membrane surface. To illustrate these distinct localization patterns and orientations, representative simulation snapshots are shown in Figure 7. These findings are consistent with previous reports on cholesterol (Róg et al., 2009) and progesterone (Atkovska et al., 2018). However, in the case of progesterone, this was only observed with POPC lipids containing a different number of saturated chains.

Together, these findings suggest that progesterone can localise just beneath the phospholipid headgroup region, with both terminal carbonyl groups oriented towards the aqueous phase. Unlike cholesterol, which has a single hydroxyl group at one end, progesterone has carbonyl groups at both ends of its steroid core, with similar hydrogen-bonding capacity (Le Questel et al., 2000), that likely contributes to progesterone’s increased positional freedom.

### 3.5 Lipid chain order and packing

**FIGURE 8:**
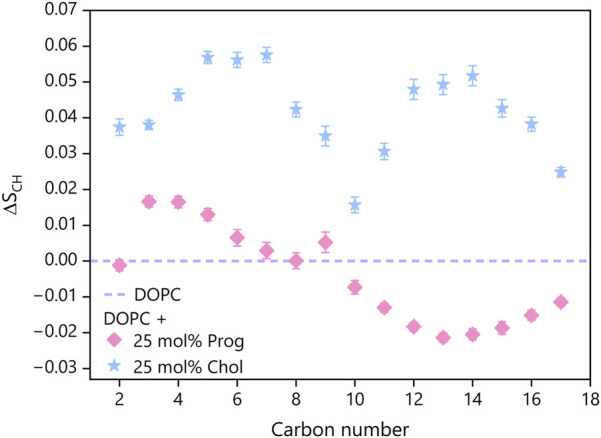
Change in order parameters ${\Delta S}_{CH}$ with respect to pure DOPC for two binary mixtures: DOPC with 25 mol% of progesterone (pink diamonds) and DOPC with 25 mol% of cholesterol (blue stars). The error bars correspond to the propagation of errors shown in Supplementary Figure S6.

To further explore how steroid positioning influences membrane structure and, in particular, to understand the molecular rationale behind the modest effect on local membrane fluidity and the pronounced effect on lipid lateral diffusion, we assessed lipid chain order using deuterium order parameter (S_CH_) for both acyl chains in DOPC membranes (Figure 8), derived from MD simulations.

Interestingly, the presence of progesterone elevated the S_CH_ values, indicating enhanced ordering, near the membrane surface (carbons 2–6), but reduced ordering in deeper regions (carbons 10–17) for both chains (see Supplementary Figure S6), compared to pure DOPC. This redistribution of membrane order implies that progesterone slightly tightens the interfacial region, while loosening the core. This differs from the uniform, substantial increase in order induced by cholesterol, which raised S_CH_ values consistently along the full length of the lipid tails, in accordance with previous reports (Ferreira et al., 2013; Yasuda et al., 2015).

To complement this analysis, we also calculated the area per lipid (APL), a parameter that reflects membrane packing and that has been widely used to analyse systems involving cholesterol (Chakraborty et al., 2020). Progesterone-containing membranes exhibited an average APL (see Supplementary Figure S7) of 61 ± (18) Å^2^, which is lower than pure DOPC (64 ± (17) Å^2^), but higher than DOPC:Chol membranes (54 ± (18) Å^2^). This is consistent with progesterone lying more horizontally within the membrane and thus occupying more lateral space than cholesterol.

These structural changes may explain the contrasting Laurdan and FRAP results: progesterone had a negligible impact on local membrane fluidity, as reported by Laurdan, but enhanced lipid lateral diffusion. The combination of increased headgroup packing near the surface and looser tail packing deeper in the bilayer may facilitate faster lipid movement without significantly altering local polarity or hydration.

## 4 Discussion

Our combined experimental and computational results provide molecular-level insights into how progesterone, a key steroid sex hormone, modulates phospholipid membranes. This highlights its potential for rapid, membrane-mediated, non-genomic actions. Using fluorescence microscopy, spectroscopic measurements, and molecular dynamics simulations, we demonstrate that progesterone influences the lateral structure of membranes, the order of lipid, and diffusion in a manner that is distinct from that of cholesterol, a membrane structural steroid.

Molecular dynamics simulations reveal that progesterone preferentially inserts itself just beneath the phospholipid headgroup region and adopts a broad range of orientations. The dominant population is aligned approximately parallel to the membrane plane. This contrasts with the rigid and upright orientation of cholesterol.

A key mechanistic difference between progesterone and cholesterol lies in their steroid core substituents. Cholesterol contains a polar hydroxyl group at the C3 position and a hydrophobic tail at the C17 position, which forces it to adopt an upright orientation within the membrane. This alignment promotes tight lipid packing and membrane thickening. In contrast, progesterone features polar keto groups at both the C3 and C17 positions, resulting in similar polarity at both ends. This structural symmetry removes a specific orientational preference, allowing progesterone to adopt a broader range of orientations, including a substantial fraction oriented more perpendicular to the membrane normal. This enables progesterone to localise closer to the lipid headgroup region by burying both polar ends near the interface.

Consequently, progesterone induces a modest increase in phospholipid order near the membrane interface, likely due to partial alignment or steric interaction with the headgroup region. This is reflected by a subtle blue shift in the Laurdan fluorescence spectrum upon progesterone addition, which suggests a minor effect on interfacial hydration and/or dipolar relaxation near the fluorophore.

However, this local ordering is counterbalanced by modest disordering deeper within the bilayer, where progesterone likely increases the free volume, allowing the acyl chains to relax. Although the magnitude of the change in order near the interface and within the core is comparable, it is likely that the disordering of the acyl chains dominates the dynamic response, resulting in enhanced lipid mobility at intermediate concentrations (10–30 mol%). FRAP measurements and MD-derived diffusion coefficients both confirm this effect in stark contrast to cholesterol, which consistently reduces diffusion. At higher concentrations (40–50 mol%), however, lipid mobility returns to, or slightly falls below, control values. This biphasic pattern likely reflects progesterone’s dual influence on lipid packing: increased order near the headgroup region (carbons 2–6) counterbalanced by decreased order in the hydrophobic core (carbons 10–17). At lower concentrations, the additional free volume facilitates lipid motion, but at higher concentrations steric crowding offsets this mobility gain. Importantly, such high concentrations are unlikely *in vivo*, even under pharmacological conditions.

Previous studies have shown that progesterone can intercalate into lipid bilayers and alter membrane properties, such as fluidity (Whiting et al., 2000; Tsuda et al., 2002; Korkmaz and Severcan, 2005; Abboud et al., 2015). For instance, Korkmaz and Severcan (Korkmaz and Severcan, 2005) investigated the effect of progesterone on fluidity of the membrane of multilamellar vesicles composed of saturated phospholipid dipalmitoylphosphatidylcholine (DPPC). By combining the data from differential scanning calorimetry (DSC), Fourier transform infrared (FTIR) spectroscopy, and turbidimetry, the authors concluded that progesterone decreases the order (increases fluidity) of DPPC bilayer at lower concentrations (3 and 6 mol%), regardless of phase state (gel and liquid crystalline), then has little to no further effect on fluidity at higher concentration (12, 18, and 24 mol%). Abboud et al. (2015) also used DSC and FTIR to evaluate progesterone-induced changes in the fluidity of the same phospholipid system and found a continuous increase in membrane fluidity with increasing progesterone content (in the range of 1–20 mol%). It is important to note that the aforementioned studies used saturated lipids such as DPPC, which retain a high degree of chain order even in the liquid-crystalline phase (Mills et al., 2008). In contrast, DOPC membranes, which are intrinsically disordered in the liquid-disordered phase, offer less space for further fluidisation by progesterone. This difference in baseline lipid order likely explains why progesterone produces measurable fluidising effects in DPPC systems but only modest changes in DOPC membranes at similar concentrations.

Indeed, both previous studies and the current work demonstrates that these effects are highly context-dependent, becoming more apparent in phase-separated, multicomponent systems than in homogeneous membranes. While cholesterol stabilises liquid-ordered domains by promoting phase separation between saturated sphingolipids and unsaturated phosphatidylcholines, progesterone impairs this process. Fluorescence microscopy reveals that progesterone leads to smaller, more irregular domains with reduced fluidity contrast, indicative of weakened lipid demixing. The broader orientation distribution and dynamic positioning of progesterone, with a dominant population lying parallel to the membrane plane, likely disrupt cooperative lipid–lipid interactions. This reduces line tension and softens the thermodynamic forces necessary for stable domain formation. Furthermore, when progesterone inserts into the lipid bilayer, it disrupts the packing of lipids by increasing the spacing between them, which introduces disorder to the lipid tails and ultimately causes the membrane to become thinner. As the bilayer becomes more disordered and less compact, its ability to act as a selective barrier is compromised. This makes it easier for ions and small polar molecules to pass through, thereby enhancing passive diffusion across the membrane.

Together, these findings demonstrate that progesterone promotes lipid lateral diffusion and suppresses large-scale phase separation, while only having a modest effect on interfacial lipid order. This nuanced biophysical behaviour may have important implications for membrane-associated signalling. Although lipid rafts and ordered domains are commonly considered to be platforms that facilitate signalling by clustering specific proteins, an increasing body of evidence suggests that it is the dynamic reorganization of these domains, rather than their static presence, that is fundamental to effective signal transduction (Lingwood and Simons, 2010; Shelby et al., 2023). By softening phase boundaries and reducing line tension, progesterone may enable more fluid reorganization of membrane nanodomains, thereby enhancing the ability of cells to rapidly assemble or disassemble signalling complexes in response to stimuli. Such increased lateral lipid and protein mobility could support the recruitment of receptors and ion channels into temporary clusters, particularly in conditions requiring rapid, reversible responses. Furthermore, changes in membrane permeability could impact cellular signalling, ion channel function, and membrane-associated protein activity. Therefore, progesterone-induced modulation of membrane phase behaviour could create a more adaptable and responsive signalling environment, particularly where rapid, non-genomic responses are essential.

Our simulations and experiments were conducted using progesterone concentrations in the tens of mol%, which exceed typical serum levels in physiological contexts (1–50 nM in non-pregnant women and up to ∼1 μM during pregnancy) (Lim et al., 2020). However, the local concentration of progesterone within membranes can be substantially higher due to its high membrane/water partition coefficient of a few thousand (Kühn-Velten, 1991; Crowley et al., 2022). Scaling arguments, such as those by Alsop et al. (2016) and reviewed by Crowley et al. (2022), suggest that even a modest systemic dose (e.g., 100 mg) can enrich membrane concentrations of progesterone to levels of ∼20 mol% or more, particularly following intramuscular injection or localised delivery. Thus, although our study models supraphysiological levels, these concentrations may be transiently achieved under pharmacological conditions.

We therefore propose that, although progesterone’s membrane-mediated, non-genomic effects may be limited under normal physiological conditions, these effects could become functionally significant during high-dose therapeutic interventions. Injectable progestins used for contraception or hormone therapy can increase the amount of steroid in the membrane to a level that can alter the structure of the bilayer and influence receptor-independent signalling.

In summary, our findings support the emerging view that progesterone acts as both a receptor ligand and as a biophysical modulator of membrane organization. By increasing lipid diffusion, disrupting phase separation, and subtly tuning interfacial and hydrophobic core order, progesterone may help to establish membrane conditions that are favourable for rapid, non-genomic signalling, particularly in pharmacological contexts where local steroid levels are elevated.

## Data Availability

The datasets generated and analysed for this study can be found in the Zenodo Repository https://zenodo.org/records/15835847, doi: 10.5281/zenodo.15835846.
