# Supplementary material for: Distinct effects of progesterone and cholesterol on lipid membranes: insights from biophysical experiments and molecular dynamics simulations
Source: Front Mol Biosci. 2025 Oct 13;12:1662811. doi: 10.3389/fmolb.2025.1662811 (PMC12554614; doi:10.3389/fmolb.2025.1662811)
Supplement: Supplementary file 1 [file DataSheet1.pdf]

Distinct Effects of Progesterone and Cholesterol on Lipid Membranes: Insights from Biophysical Experiments and Molecular Dynamics Simulations

Anna Łągowska[1*], Emilia Krok[1], Maria Domanska[2], Piotr Setny[2], Lukasz Piatkowski[1], Hanna Orlikowska-Rzeznik[1*]

[1]Institute of Physics, Faculty of Materials Engineering and Technical Physics, Poznan University of Technology, Poznan, Poland
[2]Biomolecular Modelling Group, Centre of New Technologies, University of Warsaw, Warsaw, Poland

***Correspondence:**
Anna Łągowska, anna.lagowska@doctorate.put.poznan.pl
Hanna Orlikowska-Rzeznik, hanna.orlikowska-rzeznik@put.poznan.pl

Supplementary Experimental Results

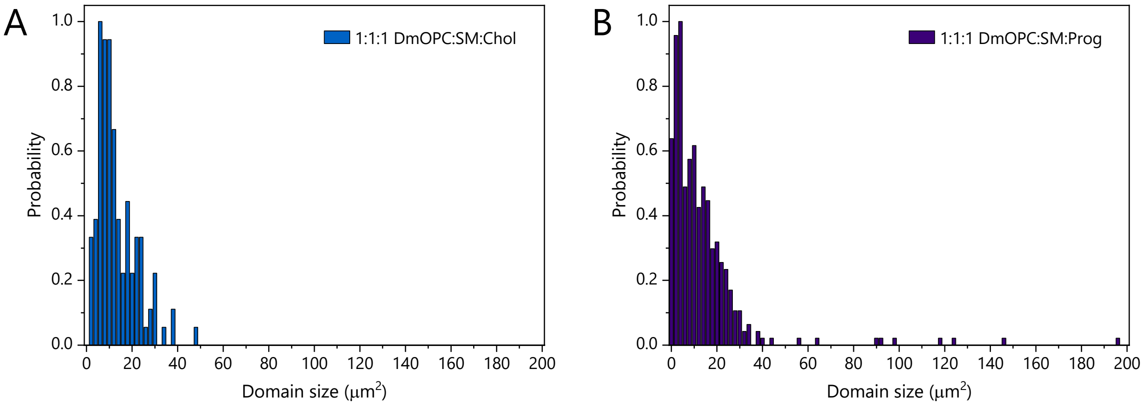

Figure S1. The histograms show the distribution of domain sizes for phase-separated membranes containing either (A) cholesterol (1:1:1 DmOPC:SM:Chol) or (B) progesterone (1:1:1 DmOPC:SM:Prog). The number of analysed domains per condition ranges from 123 to 353. Only domains that were clearly above the diffraction limit (i.e. with a diameter of over 3 μm) were included in the analysis. These calculations were performed using the Fiji/ImageJ software.

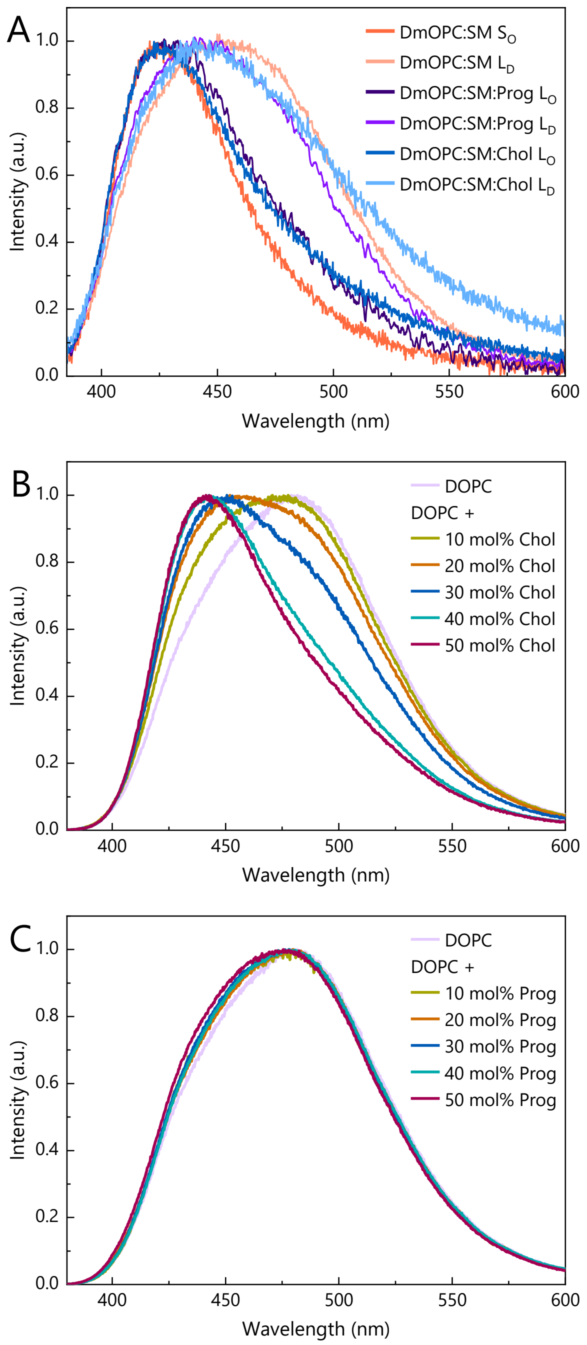

Figure S2. Laurdan fluorescence emission spectra obtained for: (A) the S_O/L_O and L_D phases of DmOPC:SM, DmOPC:SM:Prog and DmOPC:SM:Chol heated membranes, (B, C) DOPC with increasing amounts of cholesterol and progesterone from 0 up to 50 mol% (the intermediate amounts are 10, 20, 30, and 40 mol%). At least 20 and 10 spectra from distinct areas were analysed for single phase and phase-separated samples, respectively. All spectra were obtained at an ambient temperature (21°C).

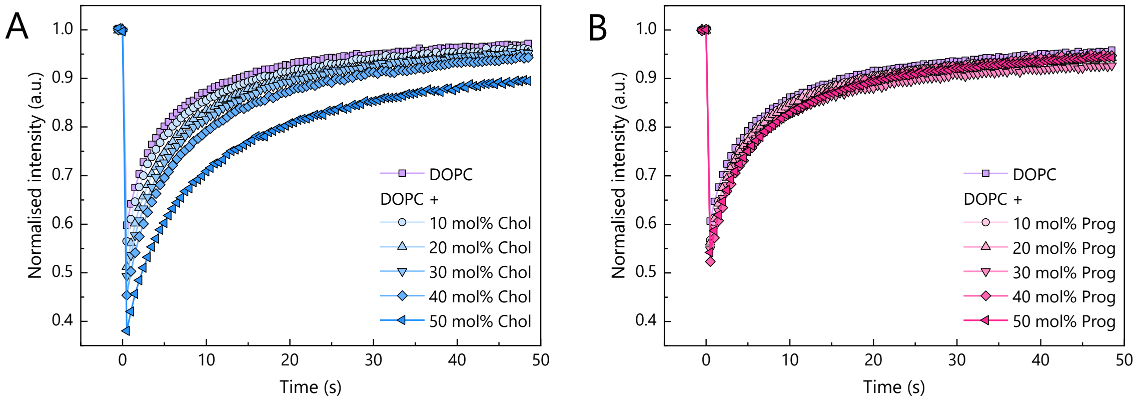

Figure S3. (A, B) FRAP traces for mica-supported lipid bilayers composed of pure DOPC and binary mixtures of DOPC and a steroid molecule at cholesterol and progesterone amounts ranging from 0 up to 50 mol% (the intermediate values are 10, 20, 30, and 40 mol%).

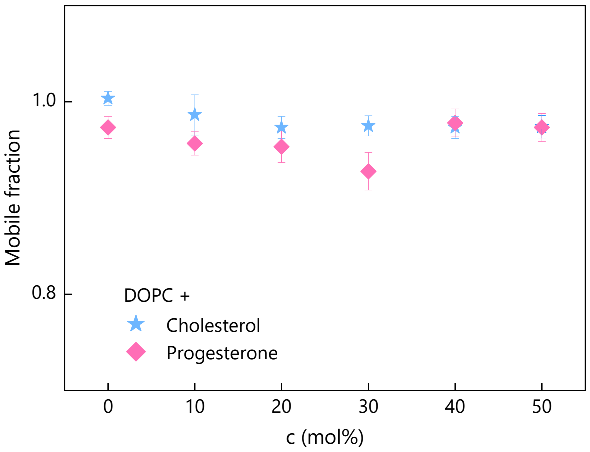

Figure S4. Mobile fractions extracted from the fits of the modified Soumpasis formula (see *Materials and methods*) to the FRAP traces presented in Fig. S3. Error bars indicate standard deviations.

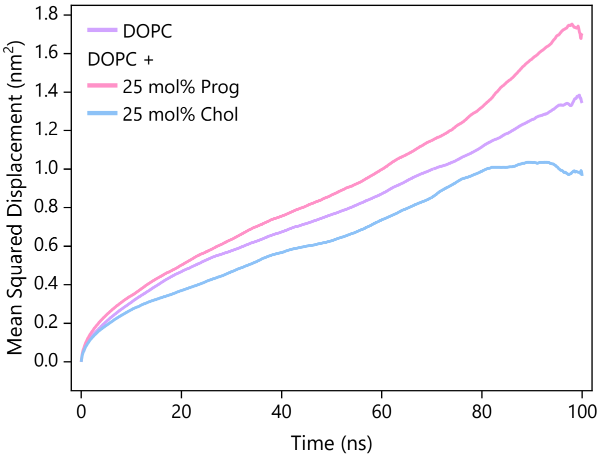

Figure S5. Mean squared displacement (MSD) of phosphorus atoms in the last 100 ns of the simulation for pure DOPC and binary mixtures containing 25 mol% progesterone or 25 mol% cholesterol.

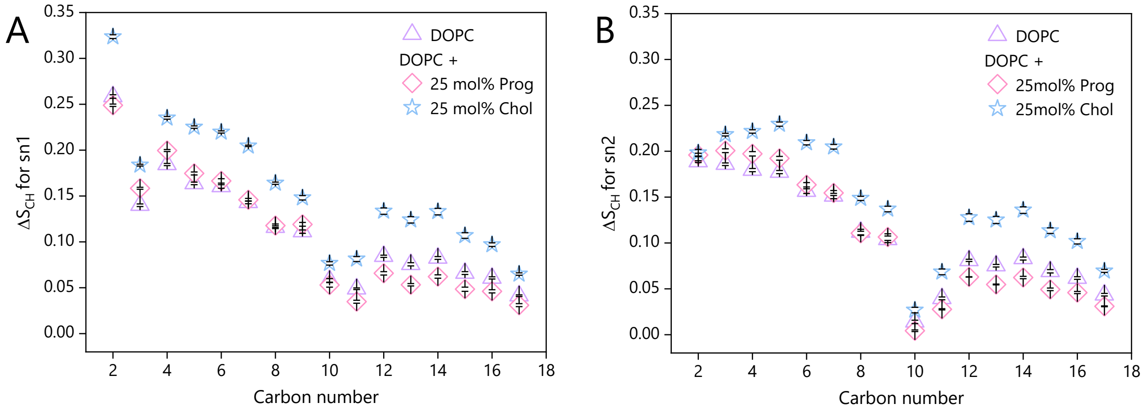

Figure S6. Order parameters for three different compositions: pure DOPC and two binary mixtures of DOPC with either 25 mol% of progesterone or 25 mol% of cholesterol, for the first (A) and second (B) chain of DOPC. The error bars correspond to the standard error of the mean estimated based on the block averaging of 5 consecutive trajectory segments from the final 100 ns of the simulation.

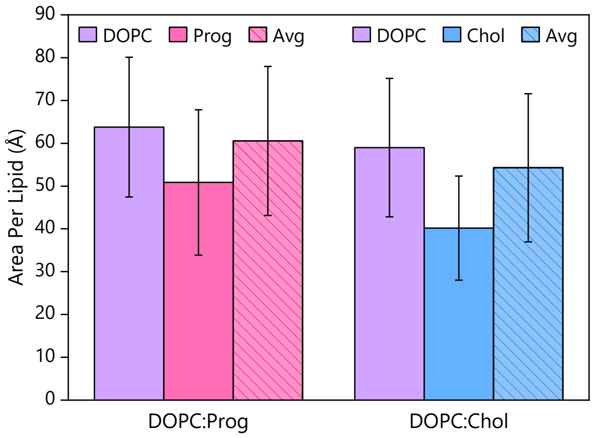

Figure S7. The area per lipid parameter for membranes composed of DOPC containing 25 mol% of progesterone or cholesterol, respectively. The lipid-specific values were obtained using Voronoi tessellation based on representative lipid atoms in the membrane plane (see the *Materials and methods* section), while the average values were calculated by dividing the membrane surface area by the total number of lipid molecules (including both the DOPC and the sterols), regardless of their chemical identity. Error bars correspond to the standard deviation of the APL distribution for the respective lipid type.

Table S1. Comparison of the data obtained experimentally for phase-separated systems.

**System** \ **Membrane composition**			**1:1 DmOPC:SM**	**1:1:1 DmOPC:SM:Prog**	**1:1:1 DmOPC:SM:Chol**
**Phase-separated membranes**	*Preparation temperature (°C)*		60	60	60
**Phase-separated membranes**	*Circularity*		0.60 ± 0.13	0.74 ± 0.11	0.84 ± 0.04
**Phase-separated membranes**	*Shannon entropy*		6.65	8.52	6.56
**Phase-separated membranes**	*Spectral centre of mass (nm)*	*S_O/L_O phase*	449.7 ± 2.4	451 ± 3	453 ± 9
**Phase-separated membranes**	*Spectral centre of mass (nm)*	*L_D phase*	466.5 ± 0.5	462.05 ± 1.1	467 ± 11

Table S2. Comparison of the data obtained experimentally for single phase systems.

Single phase membranes				
Membrane composition	*Preparation temperature (°C)*	*Spectral centre of mass (nm)*	*Diffusion coefficient (μm²/s)*	*Mobile fraction*
**DmOPC + 30 mol% Prog**	21	472.30 ± 0.23	–	–
**SM + 30 mol% Prog**	21	447 ± 6	–	–
**DOPC (I series)**	21	482.57 ± 0.16	1.68 ± 0.09	0.973 ± 0.012
**DOPC + 10 mol% Prog**	21	480.97 ± 0.07	1.96 ± 0.12	0.957 ± 0.013
**DOPC + 20 mol% Prog**	21	481 ± 1	1.91 ± 0.14	0.953 ± 0.017
**DOPC + 30 mol% Prog**	21	480.9 ± 1.1	1.89 ± 0.09	0.93 ± 0.02
**DOPC + 40 mol% Prog**	21	481.4 ± 0.7	1.68 ± 0.12	0.978 ± 0.015
**DOPC + 50 mol% Prog**	21	479.6 ± 1.2	1.6 ± 0.1	0.973 ± 0.015
**DOPC (II series)**	21	482.57 ± 0.16	1.83 ± 0.07	1.004 ± 0.008
**DOPC + 10 mol% Chol**	21	479.58 ± 0.62	1.77 ± 0.11	0.986 ± 0.021
**DOPC + 20 mol% Chol**	21	477.05 ± 0.43	1.70 ± 0.14	0.973 ± 0.012
**DOPC + 30 mol% Chol**	21	474.2 ± 0.3	1.50 ± 0.12	0.975 ± 0.011
**DOPC + 40 mol% Chol**	21	467.68 ± 0.14	1.39 ± 0.11	0.973 ± 0.012
**DOPC + 50 mol% Chol**	21	465.6 ± 0.6	1.05 ± 0.14	0.974 ± 0.012

Table S3. Direct comparison of the data obtained experimentally for single phase systems and the corresponding molecular dynamics studies.

**System** \ **Membrane composition**		**DOPC**	**DOPC + 25 mol% Prog**	**DOPC + 25 mol% Chol**
**Experimental single phase membranes data**	*Preparation temperature (°C)*	21		
	Spectral centre of mass (nm)	482.57 ± 0.16	–	–
	Diffusion coefficient (μm²/s)	1.75 ± 0.11	1.90 ± 0.11	1.58 ± 0.16
**Molecular dynamics simulation data**	*Lipid composition*	240	180 + 60	180 + 60
	Water molecules	10994	9960	9960
	Temperature (°C)	24.85		
	Simulation time (ns)	500		
	Analysis time (ns)	Last 100		
	Membrane thickness (Å)	38.3 ± 0.1	36.5 ± 0.3	41.6 ± 0.2
	Diffusion coefficient (μm²/s)	2.6 ± 0.7	3.1 ± 0.9	2.3 ± 0.9
	Area per lipid (Å)	64 ± 17	61 ± 18	54 ± 18
